# Review of the Quality of YouTube Videos Recommending Exercises for the COVID-19 Lockdown

**DOI:** 10.3390/ijerph19138016

**Published:** 2022-06-30

**Authors:** Alvaro Manuel Rodriguez-Rodriguez, Maria Blanco-Diaz, Marta de la Fuente-Costa, Sergio Hernandez-Sanchez, Isabel Escobio-Prieto, Jose Casaña

**Affiliations:** 1 Exercise Intervention for Health Research Group (EXINH-RG), Department of Physiotherapy, University of Valencia, 46010 Valencia, Spain; alvaro.manuel.rodriguez@gmail.com (A.M.R.-R.); josecasana@uv.es (J.C.); 2 Physiotherapy and Translational Research Group (FINTRA-RG), Institute of Health Research of the Principality of Asturias (ISPA), Faculty of Medicine and Health Sciences, University of Oviedo, 33006 Oviedo, Spain; fuentemarta@uniovi.es; 3 Center for Translational Research in Physiotherapy, Department of Pathology and Surgery, Physiotherapy Area, Miguel Hernandez University, 03550 San Juan de Alicante, Spain; sehesa@umh.es; 4 Departamento de Fisioterapia, Facultad de Enfermería, Fisioterapia y Podología, Universidad de Sevilla, 41009 Sevilla, Spain; iescobio@us.es

**Keywords:** sedentary behaviors, exercises, World Health Organization, COVID-19, YouTube, physical activity, lockdown, health promotion

## Abstract

Background: The world is experiencing a pandemic caused by COVID-19. Insufficient physical activity can increase the risk of illness. Trying to replicate a normal search that any user/patient could do in YouTube, the objective of this study was to evaluate the quality of YouTube videos related to home exercises during lockdown and their adherence to World Health Organization (WHO) recommendations. Methods: A simple search was carried out on YouTube. The first 150 videos were selected. After applying exclusion criteria, 68 videos were analyzed and evaluated. Two statistical analyses based on machine learning techniques were carried out. Videos were classified according to principal component analysis (PCA) models as ‘Relevant’ and ‘Non-Relevant’. Popularity was assessed using the video power index (VPI). Information’s quality and accuracy were gauged using the DISCERN scale and global quality score (GQS). Reliability and credibility of information that can be found on health-related websites was assessed using the Health On the Net Code (HONCode). Exercises were evaluated according to WHO recommendations. Results: DISCERN, HONCode, and GQS scored a mean of 2.29, 58.95, and 2.32, respectively. The PCA calculation allowed videos to auto-classify into high- and low-quality videos. Conclusions: The quality of YouTube videos recommending exercises during lockdown is low and doesn’t reflect WHO recommendations. Effective strategies and tools capable of indicating the quality of this information are needed to filter out erroneous or non-rigorous information that may affect people’s health. These tools should help any user/viewer to distinguish videos of high and low quality.

## 1. Introduction

By the end of 2019, a novel coronavirus known as SARS-CoV-2 (COVID-19) suddenly arose in Wuhan, China [1]. This virus manifests as pneumonia due to the fact that it attacks the lower part of the respiratory tract in humans [2]. An international public health emergency was declared on 31 January 2020. As of 14 April 2022, COVID-19 caused over 500,186,525 confirmed cases and over 6,190,349 deaths after being spread worldwide [3].

Most countries have adopted mandatory home lockdown policies. However, prolonged periods of time at home can make staying physically active a major challenge [4]. WHO defines physical activity (PA) as any movement of the body produced by its muscles that requires energy consumption, including exercise and other activities that include physical movement and are conducted as part of play, work, active transport, household chores, and leisure activities [5]. Self-quarantine and prolonged stays at home could be sources of added stress and could also pose challenges for citizens’ mental health, contributing to anxiety and depression symptoms [6], as well as increase other health risk behaviors. During the COVID-19 [7] lockdown, a proactive health strategy should be focused on avoiding sedentary behavior. In addition, insufficient PA constitutes the fourth most important risk factor for mortality (6% of deaths worldwide) and severely influences the prevalence of non-communicable diseases (for example, cardiovascular diseases, diabetes, or cancer). It also has other risk factors, such as hypertension, blood glucose excess, or becoming overweight [8]. In fact, insufficiently active people have an increased risk of death of 20% to 30%, compared to people who are active enough and have worsened wellbeing and quality of life [4].

Very few guidelines targeting public health in general consider PA routines on a daily basis for people that live in different levels of isolation during the COVID-19 pandemic [9]. Home exercise, through simple, safe, and easily implementable exercises, is well recommended to maintain good fitness levels. These exercises should include, but not be limited to, activities for strengthening, stretching, and improving balance and control and/or a combination of them [10]. WHO recommends, in a guide published on its website, for confined people without any respiratory illness or suspect of it, 150 min a week of moderated-intensity PA or 75 min a week of higher-intensity PA, or both options combined. In addition, WHO recommends to “*Follow an online exercise class. Take advantage of the wealth of online exercise classes. Many of these are free and can be found on YouTube. If you have no experience performing these exercises, be cautious and aware of your own limitations*” [4].

The use of exercise videos and eHealth, oriented to the encouragement and the delivery of PA through internet, social networks, TV, and mobile technologies are feasible options to maintain mental health and physical fitness during these important periods [10]. As of 3 March 2020, an estimated 58.7% of the world population had access to the internet [11]. In this situation, YouTube attracts 95.0% of all internet users. With much more than 30 million daily users, it is the biggest video website in the world [12]. It has 122 million daily active users, 1 billion hours watched daily, and it attracts about 44% of all internet users [13]. However, there is no data about the quality of the available eHealth and exercise videos, which is particularly relevant in this lockdown period due to COVID-19. Uploaded videos with health content coming from different sources present the risk of showing misleading and inaccurate information to users [14], and the authorship, the quality, and the validity of the information in the videos are essential topics to be considered [12]. In fact, many healthcare professionals and different public authorities have expressed their concerns about the quality and the veracity of the information that can be found on this website.

The objective of this study was to evaluate the quality of YouTube videos related with home exercises during lockdown and their adherence to World Health Organization (WHO) recommendations.

## 2. Materials and Methods

### 2.1. Search Strategy

On 3 November 2020 a search on https://www.youtube.com (accessed on 3 November 2020) was carried out using this specific search term: “*COVID Exercises Home*”. Our aim was to evaluate the quality of YouTube videos related to home exercises during lockdown and their adherence to WHO recommendations after replicating a simple search process that could be performed by any individual user. No filters were applied in order to avoid limiting the search, so YouTube was permitted to sort video results by relevance in accordance to its ranking algorithm active on that particular day.

The 150 videos that appeared in first order for viewers were considered for this study. They were listed in a spreadsheet for coding them (using the video URLs) and submitted to a duplicates screening (21 duplicated videos were excluded). The exclusion criteria were also applied by the researchers.

**Figure 1:**
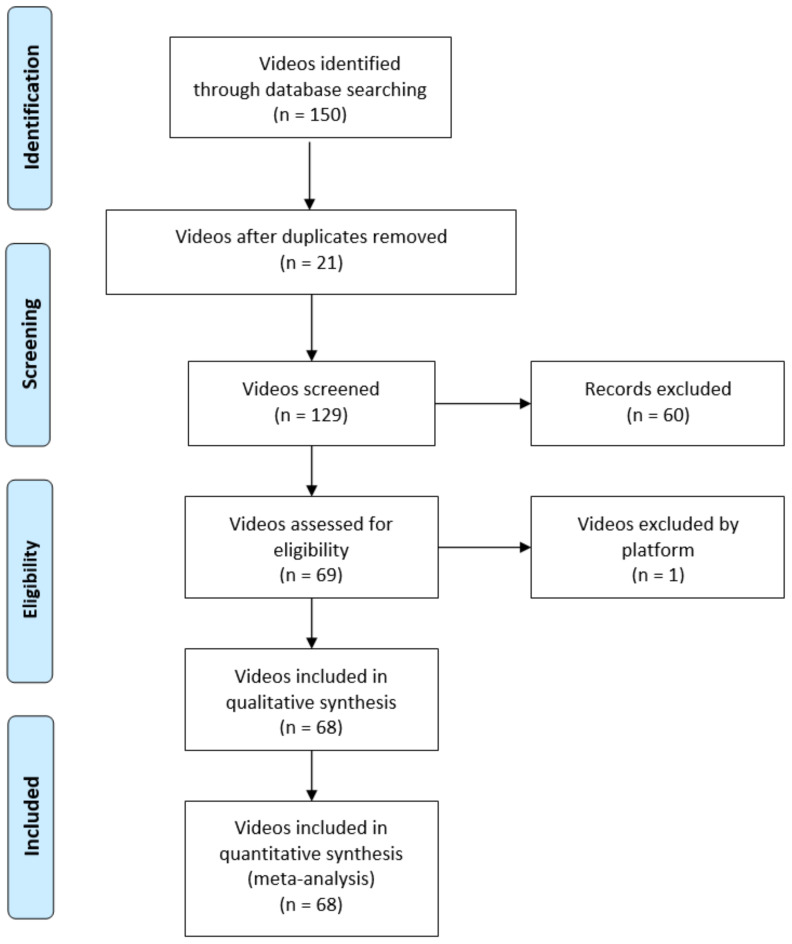
Flow diagram. From [15].

The exclusion criteria were applied to the 129 videos that remained after duplicates were removed. These exclusion criteria were: (a) non-English language videos were removed; (b) videos that didn’t show exercises were not considered; (c) videos with advertisements were also removed. During the analysis, one of the videos was removed by the platform, so the final result was 61 excluded videos. Finally, 68 videos were independently viewed, analyzed and evaluated by two different researchers (Figure 1). These examiners were each members of a research group at their corresponding universities with long and intensive experience in health topics research.

### 2.2. Outcome Measures

For each video, its descriptive characteristics were collected, such as number of views (view counts), likes and dislikes received, continent of origin, number of days online and their publication date, the author of the video, and its duration (length in minutes and seconds).

Based on their author (source of production), videos were classified into seven groups: health organizations (hospital/clinic), healthcare workers, non-healthcare workers, academic institutions, media (newspaper, TV...), non-governmental organizations (NGO), and sports institutions.

The data referring to the type of target audiences were collected as children, adults, elderly (third age), or all of them, and it was noted if the user had any previous pathology.

Videos were also codified according to their continent of origin (Australia, America, Africa, Asia, and Europe), if the exercises required any kind of material (help/support) to carry them out (professional or home), and the type of exercise (physical, psychic, or physical and psychic).

Exercise time was calculated by multiplying days per week and the recommended exercise time on each video and was classified according to meeting WHO recommendations for exercises during lockdown (150 min/week of moderate exercise, 75 min/week of vigorous exercise or both combined) [4]. The number of exercises contained in each video that agreed with the 12 exercises during lockdown recommended by WHO were also collected [4].

Video popularity was determined by the use of the video power index (VPI) (likes count/(dislikes count + likes count)) × 100)] [12,14,16] and the view ratio (VR) (view counts per days online) [17].

The reliability and the educative quality of the 68 selected videos were evaluated by the DISCERN [18] scale and the global quality scale (GQS) [19]. A modified five point DISCERN tool [20] was used, which was adapted from the original. It consists of 5 different questions, and one point will be received if the video fulfills that topic or zero points if it does not. The original questionnaire known as the “Quality Criteria for Consumer Health Information” was developed by the “Public Health and Primary Care Division” of Oxford University (London) to assess the information quality of treatment choices regarding health issues, and it was published for the first time in 1999 [21]. DISCERN scores between 4 and 5 points are sorted as “Very High”, between 3 and 4 as “High”, between 2 and 3 as “Average”, between 1 and 2 as “Low”, and “Very Low” between 0 and 1. Higher scores in the scale indicate higher levels of quality of the information [21].

GQS evaluates the overall quality of resources that can be found online. Each of the five criteria that can be identified in a video can receive one point, 5 points being the highest educational quality [19]. GQS incorporates the quality of the information and its accessibility, the general information flow, and how helpful it would be for any user [17,22]. The classification scale used was the same as for the DISCERN scale (from “Very Low” to “Very High”).

All the videos were also evaluated with the HONCode tool, which was developed by the Health on the Net Foundation [23,24], a nonprofit organization accredited by the United Nations, who elaborated the code of conduct in order to standardize the reliability of online medical/health information [24,25]. HONCode is the most frequently used assessment tool [26] for reliability and credibility of the information that can be found on health-related websites and its certification is submitted to an annual review process made by the HON-Foundation, who also responds to any violation reported by internet users.

The HONCode is not aimed to grade the information quality that is contained in a website, but rather establishes a series of rules in order for website publishers to comply with basic ethical standards of information delivery and to help ensure that visitors are always aware of the purpose and the source of the data they are viewing. It is constituted by a group of parameters about the reliability and credibility of the information that can be found in health-related websites. It is based on 8 principles determining the reliability of web pages and it can score from 0% to 100% [25].

The mentioned principles are as follows: principle 1 is about “*Authority*” and it checks the authors’ qualifications; principle 2 checks the “*Complementarity*” regarding the information to support and not to replace; principle 3 is about “*Confidentiality*”, regarding the respect the site users’ privacy; principle 4 checks the “*Attribution*”, i.e., the citation of the dates and sources of the medical information; principle 5 is about “*Justifiability*”, that is, the justification of claims and if they are balanced and objective; principle 6 checks the “*Transparency*” regarding accessibility and the delivery of valid contact details; principle 7 is about “*Financial disclosure*”, and it checks if funding details are provided. Finally, principle 8 checks the “*Advertising*”, i.e., if it distinguishes advertisements in a clear way from the editorial matter [25].

### 2.3. Statistical Analysis

On the dataset, two different statistical analyses were carried out: The first of them, which was modeled on techniques of machine automated learning, considers a problem of binary classification. It divides, using principal component analysis (PCA) [27], the dataset into two groups: “C1” or “*Relevant Videos*” and “C2” or “*Not Relevant Videos*”. With PCA, the main information in a set of data can be visualized and described by multiple and interrelated variables. The information of any dataset is matched with the total variation. So, PCA finds some directions (known as principal components) where the variance is maximized in the data. It is very useful for extracting the most important information from a set of data and it expresses the information referred to the new group of variables (or principal components). With this, PCA shrinks the number of dimensions of a set of data to a smaller number of dimensions or principal components. For this study, two dimensions were enough to bundle the data to be represented graphically, with a minimal forfeiture of the information of the original dataset.

The aim of the first statistical test is to establish which variables distinguish in the best way the groups into which videos are divided. The matter of this classification was checked in three situations according to these variables:

- DISCERN: Videos classified in the “*C1/Relevant Videos*” group are the samples whose scores were bigger than the mean; the opposite was defining the second group, “C2”.
- Exercises number: Comparable with the preceding variable.
- PCA: The binary variable defines both groups after a principal component analysis with a k-means algorithm. With this, C1 and C2 groups are defined by information variability with no loss of previous information. Accordingly, no information is lost when the dimensions are reduced to only two PCA, in this case.

For these analyses, a grouping algorithm was used to obtain the least number of dimensions that best divides the videos into two different clusters. Precision was established by *Leave-One-Out Cross Validation* (LOOCV) with the use of the “Nearest Neighbor Classifier”. Fisher’s ratio (FR) was used to determine the variables’ power of discrimination. The higher the Fisher’s ratio is, the more discriminatory they are, which is a consequence of their low intraclass dispersion and their high interclass distance. In the case of a binary classification, the FR of a variable “*j”* is determined by:

$$\frac{{\left(\mu_{j1}-\mu_{j2}\right)}^{2}}{\sigma_{j1}{}^{2}+\sigma_{j2}{}^{2}}$$

where $\mu_{ji}$ is the measurement of the mass center of the distribution of probability of the variable “*j*” in group “*i*” (*i* = 1, 2), while $\sigma_{ji}$ is the measurement of the dispersion inside that group.

Apart from FR, the Spearman, Kendal, and Pearson correlation factors were also calculated for them according to the determined classes. These additional factors disclose the importance, or discrimination power, of these variables according to the classification criteria.

The second statistical analysis used the Wilcoxon test and the *t*-test, showing the significance of the differences among both groups according to each variable’s point of view. This second test determines how relevant the variables are according to the v classification of the videos within group “C1” and group “C2”, for all three situations. A *t*-test establishes whether the mean of a variable in a group has a significant difference with respect to the other. The H0, or null hypothesis, considers no such difference among these groups. If the result of the test is 1, it shows a big enough evidence that it rejects the H0 hypothesis, while if the result is 0, such H0 will be accepted. The accepted level of error probability or statistical significance is α < 0.05. Regarding the Wilcoxon test, it determines the difference among every set of couples and it tests this difference. According to this, its H0 or null hypothesis considers the equivalence between population medians for both videos’ groups “C1” and “C2”.

According to principal components analysis, Henri Kaiser (1970) presented a measure of sampling adequacy (MSA) for factor analytic data matrices that was subsequently adapted by Kaiser and Rice (1974). It is the function of the square of the elements of the matrix when they are compared to the original correlations’ squares. This factor, renowned as the Kaiser–Meyer–Olkin (KMO) index, is considered as “Unacceptable” if it is under 0.50, “Miserable” among 0.50 and 0.60, “Mediocre” if it is more than 0.60 and less than 0.70, “Middling” among 0.70 and 0.80, “Meritorious” for 0.80 to 0.90, and if it is more than 0.90 (and less than 1.00) it is classified as “Marvelous” [28]. The KMO test was used to determine the multivariate normality and the sample adequacy. With the objective of evaluating the validity of the construct, the sample suitability for factor analysis was made using Bartlett’s test of sphericity [29]. Additionally, the statistical power of the statistical analysis carried out was checked through the combination of the PCA analysis and the KMO index.

DISCERN, HONCode, GQS, and number of exercises, regarding PCA1, show a Pearson correlation factor of 0.867, 0.791, 0.964, and 0.504, respectively, with a *p*-value < 0.01, which denotes a high statistically significant relation.

For the assessment of the concordance between examiners, the intraclass correlation coefficient (ICC) analysis was performed with confidence intervals (CI) of 95% considering a two-way random model, mean rating (k = 2) consistency, and the method of Pearson’s correlation. The significance level was fixed at *p* < 0.05. With a confident interval of 95%, values resulting from the ICC calculation under 0.5 mean a “Poor” reliability, “Moderate” for values between 0.5 and 0.75, “Good” between 0.75 and 0.90, and “Excellent” reliability for values higher than 0.90 [30].

## 3. Results

### Characteristics of Videos

The majority of the analyzed videos were created by non-healthcare workers (47%) and sports institutions (25%). Those produced by healthcare workers and media share a percentage of 9%, respectively, academic institutions reached 6% and, finally, videos produced by health institutions reached 4% of the total. As for the origin, 63% of the videos were made in America, followed by Europe (19%), Asia (15%) and, finally, Africa and Australia with 1% each.

The target audiences for which the videos are intended are mostly adults (80.9%). We found that videos intended for children reached only 1.5% of the total, 5.9% for the elderly, and 11.8% for all audiences. Of the total, only 1.5% of the videos are intended for users with some type of pathology. To carry out the exercises, the authors do not use any type of material in 39.7% of the videos, in 29.4% of them some kind of domestic material is used, and in 30.9% of the cases the author uses professional material. The data collected regarding the exercises’ time show that in 75.0% of the videos, they did not adjust to the time recommended by WHO for this type of activity, compared to 25.0% of them that did adjust to it. The inter-reviewer agreement for this study scored 0.9299, which points to the concordance between both examiners for a categorization as “Excellent”. The Kaiser–Meyer–Olkin measure for the adequacy of the sample (KMO index) for the PCA was performed to check the sample size compliance before applying the analysis of factors. The KMO measure is a test that is used to decide whether a group of samples are suitable for conducting factor analysis, and it is calculated in terms of the correlation and partial correlation between the variables. The result of this test showed that the KMO value reached 0.845. According to this, it can be determined that the size of the sample was, according to Kaiser, “Meritorious” for this dataset. This KMO measure also indicates the power of statistical analysis that was carried out.

The sphericity test of Bartlett (BST) [29] was performed to determine if there is an intra-correlation between the dataset variables considering partial correlations. It unveiled that there was a statistically significant relation (χ^2^ = 142.577; *p* < 0.001), so the sample had a good adequacy for factorized analysis, the data shows a good appropriateness for statistical-related assumptions according to multivariated normality and the data were not comprising an identity matrix. So, it can be concluded that, due to the fact that the calculated X^2^-test resulted as significant, the data matrix could be determined as appropriate. The applicability of the construct was confirmed due to the fat that the varimax rotation method and the extraction method were combined in the PCA analysis. The total variance explained with this PCA analysis was 85.507%.

The data used for each video were the mean of the scores assigned by each of the independent researchers, including the HONCode, GQS, and DISCERN scores. According to these scores, the DISCERN mean was 2.29 (SD 0.71), HONCode scored a mean of 58.95 (SD 12.89), and GQS had a mean of 2.32 (SD 0.86). Descriptive statistic basic data of the mean results for HONCode, GQS, and DISCERN are shown in Table 1.

Regarding the scores of the videos in the different questions that DISCERN applies (considering mean ± SD), it is remarkable that question 1 scored highest (0.93 ± 0.17); followed by question 3 (0.77 ± 0.36) and, then, question 5 (0.25 ± 0.32). The lowest scoring questions were question 4 (0.24 ± 0.33) and question 2 (0.10 ± 0.28). Overall results were 2.29 ± 0.91 with a median of 2.25 (min 0.50; max 4.50).

Regarding the mean scores for DISCERN, videos’ reliability performed in 27.9% of the cases as “Very Poor”, in 45.6% of them as “Poor”, 17.6% as “Average”, 8.8% as “High”, and there was no video with a “Very High” score. However, referring to the mean GQS, the videos’ quality performed in 22.1% of the cases as “Very Poor”, in 54.4% as “Poor”, in 14.7% as “Average”, in 8.8% as “High”, and no “Very High” videos were found.

The coefficients of Pearson’s correlation for all variable’s referred to DISCERN score were also calculated. These calculations show the discrimination power (relevance) of the rest of the indicators considering a level of significance of <0.05 (Table 2).

Regarding the relation between the author and the videos’ reliability, considering DISCERN scores, videos produced by healthcare workers show the highest scores (3.58), followed by academic institutions (3.25), health institutions (2.83), media (2.50), sports institutions (2.41) and, with the lowest score, non-healthcare workers (1.78). Similarly, according to the other variables referring to educational quality, GQS, and videos whose authors are healthcare workers also showed higher scores (3.50), followed by health institutions (3.33), academic institutions (3.63), sport institutions (2.44), media (2.42) and, finally, non-healthcare workers (1.84). This data (with the corresponding SD of each of them) is showed in Table 3 with the views ratio scores too. It also can be seen in Table 3 that the top scores in GQS and DISCERN scales were found in videos whose origin was Australia (3.50 in both cases), followed by those produced in Europe (2.58 and 2.54, respectively), Asia (2.25 and 2.30), America (2.20 and 2.24), and Africa (2.00 for both scales).

Regarding the five classes in which DISCERN distinguishes the videos, their VPI scores were as follows: videos that scored “Very Poor” in DISCERN had a VPI score of 97.37, videos that scored “Poor” in DISCERN had a VPI of 97.96, videos that scored “Average” in DISCERN had a VPI score of 96.53, videos that were “High” in the DISCERN score had a VPI score of 95.61, and there were no videos classified as “Very High” for DISCERN. No correlation with statistical significance was found between GQS-VPI and between DISCERN-VPI scores (*p* = 0.194 > 0.05 for DISCERN and VPI; *p* = 0.270 > 0.05 for GQS and VPI).

Referring to the number of exercises coinciding with those recommended by WHO (12 exercises), no video contains more than 5 exercises; out of those 12, only 1% of them contain 4 and 5 exercises, respectively, 25% contain 3 exercises, 26% contain 2, 31% contain 1 exercise, and 15% of the videos do not contain any of the recommended exercises. That is, the videos that contain two or less exercises represent 72% of the total and the videos that contain more than two exercises represent 28% of the total. Videos with two or less exercises obtain a DISCERN mean of 2.02, a HONCode mean of 55.52, GQS mean 2.08, views ratio mean of 685.43, likes ratio mean of 22.23, dislike ratio mean of 0.30, and a VPI mean of 98.06. Videos that include more than two exercises receive a DISCERN mean of 3.00, HONCode mean of 67.79, GQS mean of 2.95, views ratio mean of 2980.21, likes ratio mean of 124.32, dislike ratio mean of 1.62, and VPI mean of 95.46 (Table 4).

**Figure 2:**
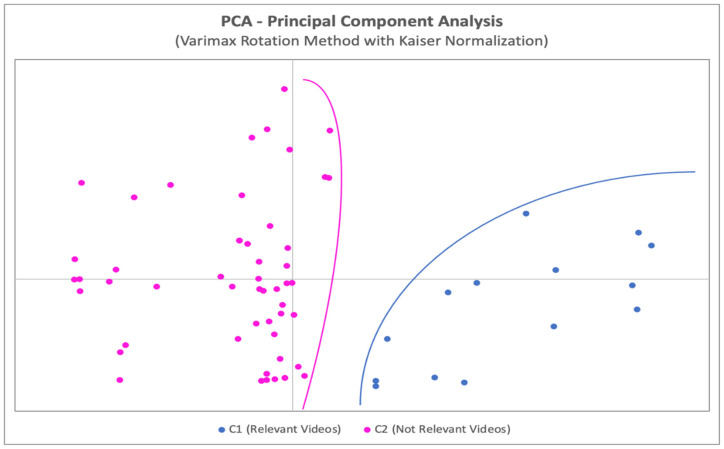
Principal component analysis graphical auto-classification of the videos.

Regarding the PCA outlook of the statistical assessment, if it is considered a graphical representation of the coordinates of each video in a coordinate system where the axes are the principal components, it can be seen that videos are grouped naturally and clearly (Figure 2), with no bias or manual intervention, in two clusters of points. These two clusters include those videos with higher scores in GQS, number of exercises, DISCERN and HONCode and in one cluster (C1), and videos with lower scores in the other cluster (C2) (Figure 2).

## 4. Discussion

The objective of this study was to evaluate the quality of YouTube videos related to home exercises during lockdown and their adherence to WHO recommendations. According to this, the main findings of the present research is that the existing videos on the YouTube platform related to PA during lockdown are of low quality and lack concordance with WHO recommendations.

Importantly, based on the data obtained, it seems that the videos related to PA during lockdown available on this platform are not produced by professionals, nor do they present reliable information since the majority of them were developed by non-healthcare workers (47%), while the academic institutions and healthcare workers only constituted 6% in both cases. This information is consistent with other research indicating that these health institutions are underrepresented in publishing videos related to health information [16] and reporting that the majority of the examined YouTube videos about breast self-examination are uploaded by individual users [22]. Regarding to DISCERN ratings, reliability and quality of most videos (73.5%) was found to be “Very Poor” (27.9%) and “Poor” (45.6%). Similar scores were obtained in the majority of videos (76.5%) for GQS (22.1% and 54.4%, respectively). There is a relation with statistical significance between GQS and DISCERN measures (Table 2). The present results highlight that caution should be taken when consuming online exercise classes in spite of the encouragement provided by the WHO in this regard.

HONCode is related to the ethics of the information presented and also with the information quality and reliability [26], which agrees with our study, that shows a relation with statistical significance between HONCode and DISCERN measures (Table 2). Additionally, videos uploaded by healthcare workers presented higher scores in GQS, HONCode, and DISCERN scales, followed by videos made by academic institutions and health institutions. These data are consistent with other studies indicating that the YouTube channels of universities produced those videos with the most precise information [31]. The lowest figures in terms of educational quality and reliability are those videos made by non-healthcare workers, which are, on the other hand, the most numerous (Table 3).

The views ratio shows the highest figures in videos that scored “High” in the DISCERN scale, which indicates that users mostly view high-quality videos (despite the fact that the quantity of these videos is low). Similar information was reported by the study of Sood et al. where helpful videos have many more visitors than deceptive videos [31]. Those videos with “Poor” quality score in the DISCERN scale achieve the highest scores in video popularity—VPI (97.96%)—while videos with “High” DISCERN score receive the lowest VPI (95.61%). These data agree with another study where videos that obtained the highest VPI scores had the lowest reliability values [32]. Moreover, according to another study, no correlation with statistical significance was identified between DISCERN, GQS values, and VPI scores [12].

Regarding the length of the videos, the results of this study agree with the literature; research videos averaged 10:37 min and preceding research found length means from 6:17 to 10:35 min [33]. Interestingly, only 25% of the videos follow the WHO recommendations regarding weekly exercise time, while 75% of the videos do not. In addition, in 39.7% of the videos, the exercises are performed without material, and in 29.4% domestic material is used. In the remaining 30.9% of the videos, the user requires some kind of technical material to carry out the exercises that does not fit with the WHO recommendations.

Analyzing educational quality and reliability, videos containing two or more recommended exercises show higher scores in DISCERN and GQS scales than videos containing two or fewer exercises, despite these being the most numerous (72% of the total). The same occurs in terms of the activity duration, since the videos that coincide in with WHO recommendations for exercise length are the best valued in these scales. That is, the videos that best fit the recommended characteristics in exercise type and completion time are the ones that obtain the best quality and reliability scores.

WHO recommends a series of 12 exercises, but none of the analyzed videos contain more than 5 of those. Of the total, 31% of the videos contain one exercise, 26% contain two exercises, 25% contain three, 15% of the videos do not indicate any of the recommended exercises, 1% contain four exercises, and another 1% contain five exercises. These figures show little coincidence between the recommendations of an organization such as WHO and the information that is available at user level in the platform that this organization specifically recommends on its website. According to Jamal et al., who systematically reviewed the effect of health information systems (HIS) on health care quality and did not find enough evidence of clinical or statistical relevant improvements in patient outcomes [34], recommendations such as those made by WHO don’t achieve the expected improvements in patients because, in this case, these recommendations are not delivered to users. Since international health organizations such as WHO recommend a series of home exercises for quarantine periods to keep people’s health in a good situation and they suggest that people can find videos on YouTube where these exercises are explained, these organizations, such as WHO in this case, should verify whether their recommendations are met on the video platforms on internet where they suggest that their recommended exercises are better explained. These recommendations are focused on being physically proactive, avoiding inactive conduct and levels of PA similar to a sedentary lifestyle. This kind of behavior could lead to adverse impacts on the health, quality of life, and well-being of people. Since WHO prepared a list of home exercises as recommendations for people in lockdown, and in these recommendations it is mentioned that they can be found on online exercises classes where many of them are free, such as YouTube, WHO should stablish some kind of control to check if their own advice is met on the platforms that they specifically mention.

Biomedical institutions and public organizations should consider if YouTube supplies viewer and (possible) patients with precise and useful data, or if its videos’ contents are possibly damaging and deceptive. These data agree with other authors stating that the data found in YouTube videos often contradict the medical recommendations and standards [35]. According to this, medical organizations and authorities should review the relevance and precision of the information that can be found on internet and should offer their help to society in accessing the most precise information [12]. All this data should be institutionally filtered. In fact, in 2001, Loretti et al. published that the WHO must improve its own performance [36], and 19 years later, with the development of eHealth and the use of the internet as one of the most broad information suppliers used, this need is even more important. The strategies necessary to develop reliable online information bring about the need to create tools that assess its quality and, although some were already developed by different scientific associations and organizations such as the American Medical Association (AMA), the Internet HealthCare Coalition, or the Health-On-the-Net Foundation (HON) [32], YouTube does not apply them despite the fact that many researchers suggest that confidence-worthy medical institutions and organizations should have more presence on YouTube and supply useful data that patients can trust [35].

After performing these two statistical analyses, the PCA calculation with the KMO index allows the videos to auto-classify into two clusters (C1 and C2, high and low quality videos, respectively) without losing any information of any variable of the study. All the variables are combined, through the PCA methodology, into two new variables that contain all the information of the initial variables. According to this, the quality of the videos can be assessed attending only to these new variables, which makes the assessment much quicker, easier, and with no lost of information, which represents an enormous advantage when performing this kind of analyses, also because the *p*-values show a relationship with high statistical significance.

The statistical strength of these two analyses is so great that it permits the self-classification of the videos, considering all their characteristics (quality among them), using only two variables (the principal components) which incorporate the data of the totality of the variables considered in this research.

### Limitations

In order to simulate standard user behavior, searches were not conducted as incognito in order to prevent the effects of geographic location and browsing history that could limit the results because some users/viewers can make this kind of modification.

This research shows the status of YouTube content at a given moment [32], due to the fact that it is a website that evolves constantly. Furthermore, the results of this study can only be considered for this website since it only takes this one into account, as mentioned in the selection criteria.

Additionally, because the study tried to reproduce the typical user’s search behavior [32], only the first 150 results were considered due to the fact that most internet visitors do not search beyond the first 50 results.

In this research, no viewers/consumer characteristics or intentions were evaluated when watching the videos. Furthermore, no video comments were considered from the comments section that YouTube adheres to each video.

## 5. Conclusions

The quality of available videos in YouTube concerning PA during lockdown is low and does not reflect WHO’s recommendations. Organizations should consider if YouTube supplies viewers and (possible) patients with precise and useful data or if its videos’ contents are possibly damaging and deceptive. Effective strategies and policies capable of indicating the quality of this information are needed to filter out erroneous or non-rigorous information that may affect people’s health. These tools should help any user/viewer to distinguish videos of high and low quality.

## Figures and Tables

**Table 1:** Descriptive statistics of the videos.

Descriptive Statistics	
(Mean ± Standard Deviation)	
Video length (minutes)	10:37 ± 10:55
View count (*n*)	59,565 ± 229,286
Days online (*n*)	42.3 ± 13.6
View ratio (Views/day)	1326.62 ± 4860.72
Likes (*n*)	2275 ± 9575
Dislikes (*n*)	29 ± 105
Likes ratio (likes/day)	50.75 ± 205.78
Dislikes ratio (dislikes/day)	0.67 ± 2.29
VPI (%)	97.33 ± 3.66
Subscribers (*n*)	238,278 ± 680,369
DISCERN score	2.29 ± 0.71
HON score	58.95 ± 12.89
Global quality score	2.32 ± 0.86

**Table 2:** Coefficients of Pearson’s correlations of each variable’s referred to DISCERN score.

DISCERN *	Pearson Coefficient	Std Error	*t*	*p*−Value **	Lower	Upper
VPI	−0.1593	0.1215	−13.108	0.1945	−0.4019	0.0833
Views per day	0.2296	0.1198	19.168	0.0596	−0.0096	0.4688
Likes per day	0.2171	0.1202	18.069	0.0753	−0.0228	0.4570
Dislikes per day	0.2234	0.1200	18.615	0.0671	−0.0162	0.4629
GQS	0.9158	0.0494	185.266	<0.001	0.8171	10.145
HONCode	0.6055	0.0980	61.813	<0.001	0.4099	0.8011
Exercises	0.5397	0.1036	52.077	<0.001	0.3328	0.7466

* Pearson’s correlation coefficients (*t*-test—2 tailed). ** α = 0.05.

**Table 3:** Author and origin variables related with DISCERN, GQS, HONCode, and views ratio.

		DISCERN		GQS		HONCode		Views Ratio	
		Mean	SD	Mean	SD	Mean	SD	Mean	SD
Source	Academic institution	3.25	0.82	3.00	1.00	66.63	19.70	280.39	156.30
	Sports institution	2.41	0.69	2.44	0.56	60.62	15.49	328.83	834.05
	Media (newspaper, TV)	2.50	0.52	2.42	0.00	58.50	11.90	2164.23	3171.15
	Health institution	2.83	0.58	3.33	1.00	72.33	12.17	272.30	343.12
	Non-healthcare workers	1.78	0.53	1.84	0.69	53.06	15.23	2172.89	6861.22
	Healthcare workers	3.58	0.82	3.50	0.82	74.25	14.51	27.31	21.07
	TOTAL	2.29	0.71	2.32	0.86	58.95	12.89	1326.62	4860.72
Origin	Africa	2.00	0.00	2.00	0.00	63.00	0.00	1907.49	0.00
	America	2.20	0.73	2.24	0.80	56.80	18.00	1532.59	5708.68
	Asia	2.25	0.48	2.30	0.74	61.85	10.15	825.62	2414.64
	Australia	3.50	0.00	3.50	0.00	69.00	0.00	60.24	0.00
	Europe	2.58	0.97	2.54	1.03	62.73	20.91	1083.46	3590.75
	TOTAL	2.29	0.71	2.32	0.86	58.95	12.89	1326.62	4860.72

**Table 4:** Descriptive statistics referred to the number of exercises shown in the videos.

		Number of Exercises		
		≤2 (0-1-2)	>2 (3-4-5)	Total
*n* (%)		72.06	27.94	100%
DISCERN	Mean	2.02	3.00	2.29
	SD	0.65	0.83	0.71
HONCode	Mean	55.52	67.79	58.95
	SD	16.68	10.33	12.89
GQS	Mean	2.08	2.95	2.32
	SD	0.72	0.92	0.86
Views ratio	Mean	685.43	2980.21	1326.62
	SD	2751.60	8156.53	4860.72
Likes ratio	Mean	22.23	124.32	50.75
	SD	100.99	358.01	205.78
Dislikes ratio	Mean	0.30	1.62	0.67
	SD	1.23	3.85	2.29
VPI	Mean	98.06	95.46	97.33
	SD	2.78	4.90	3.66

## Data Availability

All data generated or analyzed during this study are included in this article. Further enquiries can be directed to the corresponding author.
